# Report of Cases in Ophthalmic Practice

**Published:** 1859-12

**Authors:** E. L. Holmes

**Affiliations:** Chicago


ARTICLE IV.

### REPORT OF CASES IN OPHTHALMIC PRACTICE.

BY E. L. HOLMES, M.D., OF CHICAGO.

(Continued from page 652.)

### RUPTURE OF BOTH EYE-BALLS BY "GOUGING."

The following is a case of great interest, and is perhaps almost without parallel in the annals of ophthalmic science. The facts in the history of the case, previous to the time it came under my notice, I simply report as related by the patient himself. I cannot, of course, vouch for their absolute correctness. The condition of the patient's eyes, however, seemed fully to confirm his statements.

An Irish laborer, aged 37, from the country, consulted me in August last regarding his eyes. He stated that in March he was knocked down by an Irishman, who seized him with each hand by the hair of the head, and forced a thumb into each eye. After this brutal attack, he was robbed and left for some time without assistance. He was unable to see, and feared at the time that his eyes had "run out," since he felt fluid passing over his cheeks from the swollen and closed lids.

He remained nearly five weeks under the care of a physician, who prescribed poultices and wet compresses locally, and saline cathartics internally. During this time he remained totally blind, and suffered severe pain in and around the eyes. At the end of about five weeks, the pain began to subside, and on opening his eyes, he could distinguish a dim glimmer of light with his left eye. Vision was not improving; but another physician recommended him to take several powders which he prepared for him. After taking these, the patient grew quite unwell; his teeth became very loose, and for nearly three months he was unable to see as well as at first. He had used no collyrium except a solution of castile soap in water.

At his first visit to me in August, both eyes appeared normal in form, and yet, on raising the upper lids, the globes seemed flattened at their upper and inner (nasal) portion. The conjunctiva of each eye was slightly congested. Both corneæ were perfectly round and transparent. The upper and inner quarter of the right iris had been torn away, leaving the pupil elliptical in form, one side being bounded by the circumference of the cornea, and the other by the remaining portion of the pupillary margin of the iris, which was drawn considerably towards the upper and inner border of the cornea. The radiating fibres of the iris appeared stretched and separated from each other, the interstices being filled, as it were, with lymph, which gave the membrane a blemished appearance. The left iris presented precisely the same peculiar aspect as regards texture; the pupil was situated at the nasal side of the iris, and was much narrower, only about one-eighth of the iris having been torn away. At the upper and inner portion of each globe was a cicatrix, in and around which, imbedded in the conjunctiva and sclerotica, were seen numerous black spots, as if small portions of the iris and the pigment layer of the choroid had become organized in these tissues. From the appearance of the irides, I suspected a displacement of both lenses, and confirmed my suspicions by the simple experiment of Purkinje.

It is well known that if the flame of a candle be placed immediately before the convex surface of the cornea, an upright flame will be produced, which appears to be situated in the anterior chamber of the eye. The anterior convex surface of the crystalline lens also produces an upright image, situated apparently behind the iris. The posterior surface (concave) of the lens produces a larger and inverted image of the flame, situated between the other two. If the candle be moved in any direction in the facial plane, the first two images will move in the same direction with it, while the other image will move in the opposite direction.

By thus placing a lighted candle before the patient's eyes, only one image could be seen, which was the one produced by the cornea,—thus showing that there were no other reflecting surfaces in the eye corresponding to those of the crystalline lens. In neither eye was the iris tremulous, as is usually the case when the lens is removed. The contraction of the fibres of the iris during cicatrization probably rendered them so tense that the ordinary motions of the eye had no effect upon it in this case, even when the support of the lens behind it was removed. For the same reason, perhaps, light had no influence on the pupil. In the left eye, at the inner border of the cornea, where the iris was deficient, were seen small white pieces of membrane, projecting towards the pupil, which resembled portions of the capsule of the lens.

An examination with the ophthalmoscope showed that the lenses were not floating in the vitreous humor, caused probably by numerous fine black particles, scattered to such an extent through it, that it was very difficult to distinguish even the larger branches of the central artery and veins of the retina; the papule of the optic nerve was scarcely visible. This condition of the vitreous humor was more especially marked in the right eye, with which the patient was able only to distinguish light from darkness. With the other eye he could see sufficiently well to conduct himself in our streets with a little assistance from his attendant. He could not, however, define the outlines of objects, placed either near his eyes or at a distance from them. This loss of vision was caused not only by the abnormal condition of the vitreous humor and absence of the lens, but probably also by injury of the optic nerve and retina.

Although the patient appeared somewhat enfeebled, he stated that his general health was perfect. I simply prescribed pot. chlo. internally, and recommended a mild astringent collyrium for the conjunctival congestion. I also advised him to use plain nutritious diet, to exercise as much as possible in the open air, and apply the cold douche daily to the closed lids.

I have seen the patient three times since my first examination. A few days ago he could see to walk any street without assistance, and could read many of the signs upon the buildings. The right eye was also fast regaining its powers of vision. The vitreous humor was found to have recovered almost entirely its transparency; the vessels of the retina and the papule of the optic nerve were distinctly seen in each eye.

When we consider how delicate an organ the human eye is, and how often it is utterly destroyed by the finest instrument in the hands of the skilful operator, it seems wonderful that a patient could suffer such terrible violence in *both* eyes, and recover, with the form of *both* organs and the power of vision so well preserved.

Mackenzie alludes to a case, reported by Mr. Dixon, in which each eye had been deprived by a blow not only of its lens but also of its *iris*. A cicatrix existed in each eye when the lens and iris had been forced through the sclerotica. The patient was able to read very well with the assistance of a perforated card and double convex lens.

It is a singular fact that a severe blow may in one instance be received on the eye without injury, and that a slight one, in another, may force the lens out of the eye, performing as it were the operation for cataract by extraction.

In the sixth number of the *London Ophthalmic Hospital Reports* (1859), is found the history of six cases in which the lens had been forced through the sclerotica and retained between this membrane and the conjunctiva. In accidents of this kind the rupture usually takes place at the upper and inner portion of the union of the cornea and sclerotica. The lens may escape entirely from the eye, or it may often be retained as a small tumor beneath the conjunctiva, which is so elastic that it does not always rupture with the firmer membrane under it.

I am permitted to refer to a case which some years since fell under the notice of Prof. Brainard. A grain of shot entering the eye of the patient at the outer angle, had passed through the globe behind the iris, and buried itself in the tissues at the inner angle. The shot had already been removed, and the wound on each side of the globe had healed, leaving a well marked cicatrix. Vision for distant objects was almost perfect. Possibly the lens had been displaced and absorbed, as in certain operations for cataract by division.

It may not here be out of place to state that "gouging" is not peculiar to America, as affirmed by certain foreign travelers. I am informed by good authority that in some counties of England and in Ireland this brutal mode of assault is not uncommon among the lower classes.

[We can assure the writer of the above very interesting paper, that "gouging" is a mode of warfare almost unknown in the British isles. An attendance for many years on some of the most extensive eye infirmaries of the empire would have afforded us ample opportunities of making ourselves acquainted with the fact, if it existed; for, if the practice of such iniquity prevailed there, we could not fail to have observed it. We cannot, however, recollect having seen in Great Britain or Ireland a case of injury of the globe of the eye that could be attributed to violence thus inflicted, and we should almost even be induced to altogether deny its occurrence there, if we had not been aware that Bidloo, in his "Opera Omnia Anatomico-Chirurgica," related, in bad Latin, the following case:—"Vidi, ante sex annos, Londini, virum robustum, cui amba oculi, horrendum in modum protuberabant: quod ad ipsam partium dispositionem, illam genuinam observavi, præterquam quod ipsis pupilla majuscula, cæsius color et insolita esset cum duritie magnitudo: visu tamen utrumque destitutum omni expertus sum; inquirens in causam, accepi, inter luctandum ab adversario suo, ira, gymnasticas, vel luctationis *Anglicanæ*, leges excedente, pollices fuisse oculorum orbitis intrusos hominemque mox, nullo inflicto vulnere, dolore correptum summo, non medicis, non chirurgis; sed visu restitisse orbum oculosque magis magisque distendi atque protuberare, quandoque cum, quandoque sine dolore." We have frequently known the lens to be dislocated by blows on the eye, and yet useful sight has been retained. Larrey has put on record a case illustrative of the amount of violence occasionally exerted, and in which vision was preserved, although the globe was burst, the lens expelled, and the vitreous and aqueous humors had escaped. There was, moreover, separation of the lower part of the cornea from its attachment to the sclerotic, protrusion of the iris, and extensive injury of the appendages of the eye. Yet, unpromising as the case was, the globe gradually refilled, the wound cicatrized, and by the aid of a very convex glass, objects becoming distinct, the man who was the subject of the injury was enabled to pursue his profession as a soldier. In cases of violence to the eye from blows, or even from pressure on its surface, in the effort to perpetrate "gouging," we should, perhaps, apprehend as much danger to vision from the supervention of amaurosis, caused by the concussion of the retina, or even its compression, as from the rupture of the textures of the organ, or even from the separation of important parts from their connections. A case is related by Beer, in which indulgence in a practical joke thus caused incurable amaurosis from mere pressure. We give it in the words of the author: "Some years ago, I was called to a man, who had previously enjoyed excellent sight, but, a short time before I saw him, had, in an instant, became totally blind in both eyes. He happened to be in a company of friends, when suddenly a stranger stepped behind him, and clapped his hands upon his eyes, desiring him to tell who stood behind him. Unable, or unwilling, to answer this question, he endeavored to remove the hands of the other person, who only pressed them the more firmly on his eyes, till at length withdrawing them so as to allow the eyes to be opened, the man found that he saw nothing, and continued ever afterwards blind, without any apparent lesion of the eyes."—Ed.]

